# “The Monkey on Your Shoulder”: A Qualitative Study of Lymphoedema Patients' Attitudes to and Experiences of Acupuncture and Moxibustion 

**DOI:** 10.1155/2016/4298420

**Published:** 2016-08-18

**Authors:** Beverley de Valois, Anthea Asprey, Teresa Young

**Affiliations:** ^1^Supportive Oncology Research Team, Lynda Jackson Macmillan Centre, Mount Vernon Cancer Centre, Rickmansworth Road, Northwood, Middlesex HA6 2RN, UK; ^2^University of Exeter Medical School, Magdalen Road, Exeter EX1 2LU, UK

## Abstract

*Background*. Lymphoedema, a distressing consequence of cancer treatment, has significant negative impact on health-related quality of life. Multidisciplinary approaches are needed to improve physical and psychosocial wellbeing. Acupuncture and moxibustion (acu/moxa), two modalities of traditional East Asian medicine, may contribute to improved outcomes for cancer survivors with lymphoedema.* Aim*. To explore how patients with lymphoedema secondary to cancer treatment perceive and experience acu/moxa treatment.* Design and Setting*. A qualitative focus group study, nested in a 3-step mixed methods observational study, was carried out in a cancer drop-in and information centre in north-west London.* Methods*. Six focus groups and one telephone interview were conducted with 23 survivors of breast or head and neck cancer, who had completed up to 13 acu/moxa treatments. Scripts were transcribed, coded, and analysed to identify salient and overarching themes.* Results*. Participants described feeling disempowered by cancer treatment and subsequent diagnosis of lymphoedema. Acu/moxa was valued for its whole-person approach and for time spent with a practitioner who cared, listened, and responded. Participants reported changes in physical and psychosocial health, including increased energy levels and reduced pain and discomfort, and feelings of empowerment, personal control, and acceptance. Many were motivated to improve self-care.* Conclusion*. Many participants who received acu/moxa treatment reported improved wellbeing and a more proactive attitude towards self-care.

## 1. Introduction 

Cancer treatments are the main cause of secondary lymphoedema in the developed world, as surgery and radiotherapy may cause damage to lymph vessels and/or nodes [1]. The resulting imbalance between interstitial fluid production and transport causes accumulation of fluid in the tissue spaces, leading to chronic swelling, inflammation, and development of fibrotic and adipose tissue [2, 3]. Treatment, which aims to limit progression, comprises individualised programmes combining intensive treatment (decongestive lymphatic therapy) with daily self-care regimens that include skin care, wearing of compression garments, self-massage, and exercise and movement [1, 4]. Currently incurable, lymphoedema requires lifelong daily management to prevent progression.

A distressing and feared consequence of cancer treatments, lymphoedema is associated with melanomas, gynaecological, prostate, breast, and head and neck cancers [5]. Amongst breast cancer survivors the reported incidence is 20–30% [6, 7], and amongst head and neck cancer survivors it is 48–68% [8]. The condition is disfiguring, disabling, and distressing with significant impacts on health-related quality of life [9–11]. The physical and psychosocial impacts on breast cancer survivors are well documented internationally [12] and less well investigated for head and neck cancer survivors [13, 14]. In addition to swelling and increased risk of infection (cellulitis), physical consequences include a sensation of heaviness, pain, discomfort, restricted mobility, and loss of function, while psychosocial consequences include psychological distress, social embarrassment, poor body image, social isolation, and financial burden. Treatment should include multidisciplinary approaches to address quality of life, as well as the complex physiological and psychosocial problems associated with a chronic condition in patients with multiple comorbidities [15].

Complementary and alternative medicine (CAM) can be included as part of a multidisciplinary approach to health care. Cancer survivors are users of CAM with European studies reporting CAM usage by 44.7% and 22.7% of breast and head and neck cancer survivors [16, 17]. People with cancer treatment-related lymphoedema also choose CAM in addition to mainstream treatment [18], with reported usage by 45% of women with lymphoedema related to breast or gynaecological cancers [19].

Acupuncture is valued for symptom improvement and improved physiological and psychological coping by people with chronic disease, including cancer [20–22]. Its use amongst people with lymphoedema has been controversial, due to concerns about skin puncture exacerbating swelling or introducing infection [1, 23]. However, a number of early stage studies report safe and promising outcomes for acupuncture [24–27] and for the combined modalities of acupuncture and moxibustion (the application of heat to stimulate acupuncture points) [28].

Building on our previous acupuncture research, which reported improvements in wellbeing and quality of life and reduced symptom burden in women undergoing adjuvant treatment for early breast cancer [29, 30], we wished to investigate whether acupuncture and moxibustion (acu/moxa) could be used in the management of lymphoedema. Following Medical Research Council (MRC) guidelines for researching complex interventions [31], our overall study was a three-step patient-centred exploratory study employing mixed methods to investigate the feasibility of using acu/moxa to promote wellbeing and improve quality of life for breast cancer and head and neck cancer survivors with treatment-related secondary lymphoedema. Step 1 comprised focus groups to assess the acceptability of acu/moxa treatment to people undergoing conventional treatment for lymphoedema; Step 2 was a clinical treatment phase in which 35 people received up to 13 acu/moxa treatments, the results of which are reported separately [23, 32].

In this paper, we present qualitative research conducted in Step 3, in which participants who received acu/moxa participated in focus groups to discuss their experience of treatment. The aims of this step were to gather data that provided an insight into participants' perceptions of the following:

- Experience of having acu/moxa treatments.
- Meaning of “wellbeing” and the effect of acu/moxa on wellbeing.
- Effect of acu/moxa treatment on their attitude to having lymphoedema.

## 2. Materials and Methods

Qualitative methods were used in order to obtain sufficient access to the meanings and perceptions of the participants in the acu/moxa study [33, 34]. The focus group method was chosen to generate lively discussion and thus to enable participants to explore and clarify their views more thoroughly than might be achieved in a one-to-one situation [35].

### 2.1. Sample

Thirty-three study participants who completed at least seven acu/moxa treatments in the clinical phase of the study were invited by letter to participate in focus groups. Twenty-six responded favourably, although not all were available to attend on the eventual dates set for the groups. The Hertfordshire Regional Ethics Committee approved the study (08/H0311/123).

### 2.2. Data Collection

Six focus groups, lasting from 42 to 72 minutes, were held in meeting rooms at the cancer treatment centre between July and September 2009.  Table 1 details the participants in each group according to gender and cancer type, shows the nonattendees and reasons for nonattendance, and lists the durations of the focus groups.

Participants signed consent forms before the groups started and confidentiality and anonymity were assured. The focus groups were moderated, transcribed verbatim, and analysed by a researcher with expertise in qualitative research methods (Anthea Asprey). A comoderator (Teresa Young) audiotaped the sessions and observed the proceedings.

A questioning route was developed by the research team, using the recommended categories: opening, introductory, transition, key, and ending questions (Table 2) [36]. The moderator adopted a sequential method [37] to elicit detailed information about the participants' experiences of taking part in the study from start to finish. The “key questions” regarding the concept of “wellbeing” and the effect of the acu/moxa treatments on this aspect of their life were introduced to the participants at approximately the midpoint of the discussion. A flexible approach to the questioning was adopted, allowing the participants to explore aspects that were important to them and to introduce any new factors. Open questions and prompts were used to explore the participants' perspectives in depth; discussion was encouraged and supported, with each participant given the opportunity to respond.

The moderator also conducted the interview, which lasted 20 minutes, using the same introduction and questions as for the focus groups.

Data collection continued until it was felt that saturation had been reached; that is, no new codes were emerging from the data.

### 2.3. Data Analysis

The scripts were analysed thematically [38] using the computer-aided qualitative analysis package NVivo® (version 2.0). The research analysis was an iterative and reflexive process to ensure that it was comprehensive and systematic and to maximise insight into the meanings conveyed by the participants [39, 40]: as coding progressed and salient issues emerged, these were incorporated and applied to both new and previous transcripts. To ensure adherence to the need for visibility and accountability in qualitative research the themes and categories developed by the researcher (Anthea Asprey) were checked by a second researcher (Beverley de Valois) [41, 42].

## 3. Results

### 3.1. Participants

Twenty-three people with upper body lymphoedema secondary to cancer treatment participated in this qualitative study (one participant, who was reluctant to join a group, was interviewed separately). The participants were aged 43–83 years with varied demographic backgrounds (Table 3). Most were female, as the majority of study participants were breast cancer survivors; however, HNC survivors were equally represented by both genders. Of particular note was the wide range of durations of lymphoedema, ranging from recent onset (6 months) to long-term chronic (11 years).

The participants' quotations below are identified using a code number for anonymity, followed by BC for breast cancer and HNC for head and neck cancer.

### 3.2. Disempowerment, Disablement, and Disfigurement

The personal experiences and circumstances of individuals often emerged unsolicited in the focus groups. Developing lymphoedema was “adding insult to injury” (018 BC), coming on top of cancer diagnosis:

*When I was diagnosed with breast cancer, it was like the worst thing ever, and I had, obviously, the radiotherapy and the chemotherapy and then I developed lymphoedema, and it was as if I'd hit rock bottom, it was horrible.* (004 BC)

Cancer treatment, for some, was a disempowering experience. Two participants compared their treatment in the NHS to being put on a “conveyor belt” or “train” and emphasised their own passive role in the process:

*I'd lost me, I'd got so used to everybody saying ‘do this, do that' because when you're diagnosed you're put on this train, they lock the doors and they don't let you off, you know. And then suddenly you arrive at the station and they say ‘go away for 3 months'. *(003 BC)

A number of participants experienced very low self-esteem, at times bordering on despair. Participants often described feeling depressed, attributing this to the chronic nature of lymphoedema and the difficulty of alleviating it:

*Because you just go through so much, the *[cancer]* treatment is so awful, and then you have all these added bits on the end you know. So it makes you feel really down and really depressed. *(013 BC)

*You get lymphoedema as a side thing, you know, it's awful. But you've got it for life, you know. *(018 BC)

*So I got really depressed by this time, I thought that nothing was going to help.* (006 BC)

Such feelings were intensified by life events including personal tragedies, such as divorce or the deaths of relatives or close friends:

*I had some terrible times … my friend passed away and then a few months later my brother passed away so I was going through hell … I think at one point I just lost the will to live … I was so down, at the lowest ebb. *(017 BC)

Lymphoedema could be disabling, affecting daily routine activities and engendering feelings of frustration and desperation. Breast cancer survivors in particular articulated this in strong terms:

*Some days I just want to rip my arm off … plus with the weight gain as well … you get really frustrated. *(013 BC)

*When you're going anywhere you think ‘oh, can I be bothered?', I mean, even doing your hair, to hold up and do things … It affects you so much … if I'd had an axe I could have cut my arm off. And I just, you know, it's just so tiring all the time to have that kind of pain. *(003 BC)

Other individuals reported experiencing fatigue, pain, and anxieties about dealing with every day challenges such as social events and the demands of work, which could leave them exhausted. Participants also reported other conditions, sometimes not directly connected with lymphoedema; these included joint pain, sleep problems, and repeated respiratory infections. Some experienced discomfort from the side effects of medications, such as adjuvant cancer treatment (including extreme hot flushes or chronic itching) or painkillers (constipation).

The disfigurement associated with lymphoedema had personal and social consequences. One head and neck cancer survivor, who experienced intermittent bouts of lymphoedema swelling, said:

*It isn't too pleasant if you see me when it's really bad, it's dreadful actually, it is just like Spitting Image … I have to admit there are times when I can't bear to look in the mirror. *(020 HNT)

The reaction of others to their lymphoedema also caused difficulties for some participants, including social embarrassment. Enquiries, particularly from strangers, were irritating reminders, leading participants to “wish nobody mentioned it” (015 BC). Sometimes, the visibility of lymphoedema was experienced as being worse than having cancer itself:

*This is worse, because … my cancer I could hide, nobody need know about it. But this is awful, you go out … I'd always bring a cardigan with me in case I get embarrassed, because I do sometimes … it was just awful. *(023 BC)

### 3.3. The Experience of Having Acu/Moxa Treatment

#### 3.3.1. Initial Expectations

Most participants said they responded positively when it was suggested they have acu/moxa treatment. For many, the suggestion had come at a time when they had reached the point where they felt it was worth trying anything that might help:

*… things couldn't get any worse, so I was willing to try anything. *(016 BC)

*… and I thought, the agony I'd suffered with cancer and all the rest of it, the chemo and the radio, I thought ‘well, this can't be any worse, can it?' So if it helps, why not try it. *(017 BC)

Only five participants had previous experience of having acu/moxa, and consequently there was a wide range of expectations. While some were positive, others had quite low expectations. One breast cancer survivor admitted to being “sceptical,” while a head and neck cancer survivor said:

*I'd never had it before, so I was apprehensive but I thought I would give it a try … I didn't feel it was going to work, to be quite honest. *(009 HNT)

#### 3.3.2. Concerns about the Acu/Moxa Treatment

For three participants, fear of needles was a concern, especially following their experience of chemotherapy:

*Needles!! *[laughter].* That was my biggest fear, was the needles … Yeah, I thought ‘oh no!' because I've made such a fool of myself over this, but obviously having chemotherapy you do get slightly, you know, a bit blasé. But I thought ‘oh no, do I really want another round of having needles put in?' *(013 BC)

Needle phobia, however, could be overcome by the desperation to alleviate symptoms:

*I must admit, as I say, I was nervous, I don't like needles, I can't look at anyone on television having an injection! It says to me how desperate I was that I would even consider having needles put into me! *(003 BC)

The cancer drop-in centre where acu/moxa treatments were given was on the hospital site where most participants had undergone treatment for cancer. For some, returning to the hospital site was challenging. One woman, who had been treated 20 years previously, found returning was “very difficult … I certainly hate being reminded” (014 BC). For another, the hospital grounds evoked many strong feelings:

*I mean I was loath, initially, to think I'm going to come back here on a weekly basis, because I really just didn't want to come back to where I had the chemo and you'd been in hospital and surgery and whatever … That nearly put me off, coming back here … I thought, oh I don't want to come back here and see the chimney, you know, and my friend died here and … I didn't want to do that. *(018 BC)

Overall, however, participants appreciated being at the cancer drop-in centre, describing it as “welcoming,” “calm,” and “relaxing.” This latter quality was seen as especially important, especially if acu/moxa were to be made an adjunct to usual treatment for lymphoedema:

*If they were going to offer it *[acu/moxa]* as part of the treatment, then they'd have to have it in that kind of setting, I think, because I found all that very relaxing. *(012 HNT)

#### 3.3.3. Valuing a Whole-Person Approach

Participants expressed gratitude for the cancer treatment they had received in the NHS. However, there were feelings that conventional medical treatment focussed on the individual parts of the body that are perceived to be diseased, to the detriment of seeing the whole person:

*When you go to the hospital and you go to anybody, they're only looking at the bit they're looking at, and nobody actually asks how you're feeling generally …. *(001 BC)

Participants articulated frustrations they experienced with this approach and contrasted it with what they perceived as the acupuncturists' whole-person approach:

*I'd been going back and forth to the doctor and felt no-one was actually listening to me, you know, how I felt. They kept saying ‘go to the chemist' and I had done and nothing was improving, and I just felt that *[name of acupuncturist]* was listening to me. And she was taking the whole, you know, looking at me as a whole, not just at the medical problems that I had, you know. *(016 BC)

Some participants were particularly impressed by the attention the therapists paid to their individual needs, both physical and emotional, and how they adapted the treatments accordingly:

*But it wasn't the same acupuncture every time anyway. So it just depended … and every week they asked how you were in relation to different things before starting and would decide on which particular treatment would be appropriate for that week … So it wasn't like a set plan that you had to have it whether you wanted it or not, it was very flexible. *(019 BC)

*She was willing to say ‘Look I think we need to move from this, what we originally said, you need to work on this emotional side as well with the acupuncture'. So she did change things as the weeks developed and then changed back when I was a bit stronger. *(018 BC)

Participants appreciated the expertise of the acupuncturists, their caring attitude, and the time spent with them. They also appreciated being listened to and responded to:

*They were both very sympathetic and listened to what you had to say and acted upon it in what was appropriate today. *(019 BC)

*… it was therapy in the total sense … I felt cared for and I felt she *[the acupuncturist]* was tuning in to parts of my body that I didn't realise were in a bit of a dilemma …. *(011 BC)

*Coming and associating with somebody who is caring about you, gave a tremendous positive vibe, that there are people who want to really try and help you, so that itself was a positive thing. *(007 HNT)

This was not appreciated by all, as this caring approach could stir up deep, buried feelings about the cancer experience:

*It's just this: I feel deeply, deeply superstitious talking about it *[cancer]* because I had it some time ago and I've been doing OK without mentioning it to anybody except the friends who knew I had it at the time … I got upset talking to *[name of acupuncturist].* This was the first time in 20 years that I got upset … it did take me back, definitely, and I found that very difficult. *(014 BC)

Nevertheless, on the whole, participants appreciated having acu/moxa and described it as a “relaxing” experience. In spite of any discomforts associated with needling, having acu/moxa was preferable to biomedical interventions:

*Well I looked forward to going to acupuncture. I mean most things you have to go through I don't look forward to going to! I've got to go in tomorrow, with a camera down the throat, I'm not looking forward to that, but with the acupuncture I felt better every time I went, so … oh yeah! I felt better going and even better when I left. *(008 HNT)

#### 3.3.4. Moxibustion

Not all the participants received treatment using moxibustion, and those who did had mixed responses to it. Some enjoyed it immensely and found it very relaxing:

*I really liked that, especially the smell and the warmth, it's incredible … But I thought the effect was brilliant … I loved having that treatment. *(001 BC)

Others were more ambivalent and regarded the method as less successful than the acupuncture:

* I felt myself being a bit cynical. It struck me as a bit sort of … gosh what do you call those … witchdoctor-ish … they put it on and set it alight and whip it off. … I would have preferred pure acupuncture. *(014 BC)

#### 3.3.5. Unhelpful Aspects of the Treatment

There were very few complaints about participating in the research study and even fewer regarding the actual acu/moxa treatment. Dissatisfaction was expressed by some participants about the treatment room and equipment. The colour of the walls of the treatment room, which was painted egg-yolk yellow, was disliked by several:

*It sounds silly, but the colour of the room, the yellow room … it's not a restful colour is it? *(003 BC)

Others reported that they found the treatment couch uncomfortable:

*… I found the bed was incredibly uncomfortable, because I've got a bad hip and it was difficult for me to lie on that bed. It seemed so hard …. *(006 BC)

The time and expense of attending for treatment was a concern for two participants:

*Yes, the travelling and the time … I don't like driving, and I found it tiring. Also stressful, I find driving stressful … and expensive, because it was when petrol was going up dramatically. And I thought ‘oh gosh, every time I come up it's costing me so much in wear and tear on the car and everything'. *(006 BC)

*Only because I run a business from home and I'm just busy all the time. And sometimes it would be the fact that I'd actually turned a full day's work away to come to the appointment. So for me, for a self-employed person, it was a commitment. *(011 BC)

A number of participants complained about the study questionnaires; the Positive and Negative Affect Scale (PANAS), which asks for responses to twenty feelings and emotions [43], was particularly disliked:

*I was slightly irritated by the constant questions and filling in, and then I used to have to justify it to myself ‘after all this, it's really good, they've got to have some feedback'. *(020 HNT)

*It's just that the last time when I was filling in this dreadful form, this was the worst part of everything! … I hate filling out forms anyway, but this, when I think there were 38 degrees of emotion, I found that so difficult, I really did. *(014 BC)

While there were no reports of adverse events of acu/moxa treatment, three participants found aspects of the study distressing. One, quoted above, found the whole experience of the study somewhat upsetting because she found it difficult to reflect on the painful experience of having breast cancer diagnosed twenty years previously. Two other participants found the need to remove their clothing distressing and discussed that they had not been prepared for this aspect of treatment:

* That came as a bit of a shock because I expected to be only taking off a part of my clothes and when she said to strip down ‘oh I don't know if I want to strip right down'. Because as I said, when my husband went for his treatment all he took off was his shirt … It came as a bit of a surprise because I wasn't prepared for it …. *(005 BC)

* Actually, were you given warning about that? Because I wasn't. *(006 BC)

* I wasn't, that's why. *(005 BC)

* I didn't realise we'd have to take all our clothes off. *(006 BC)

Regarding the acu/moxa treatments per se, some participants expressed disappointment that their first experience was quite unremarkable:

*In the beginning, I was wondering ‘Am I wasting my time coming up here because nothing has changed.' *(005 BC)

*Well the first time … she just did a little bit of acupuncture on my back and I did not feel it really, I must admit. *(022 BC)

*Um, I think I thought to myself ‘oh, is that all there is to it?'. Because I'd only had the needles in for a short time. *(011 BC)

These participants, however, gave very positive accounts of subsequent treatments, and overall, most people had positive reports about participating in the research. Many of those who experienced difficulties with the setting, the travel involved, the discomfort of treatment, or the completing of questionnaires still reported deriving benefit from the experience.

### 3.4. Concepts of Wellbeing

The primary purpose of this study was to ascertain whether acu/moxa could be used in lymphoedema management to promote wellbeing and improve quality of life. The treatments aimed to improve overall physical and emotional wellbeing, to reduce the impact of disabling symptoms, and to improve adherence to self-care. In the focus groups, questions about wellbeing began with an exploration of how participants defined the concept.

Three individuals articulated their thoughts very clearly about wellbeing, seeing it in terms of their general attitude to life:

*Wellbeing is just waking up in the morning feeling that I can carry on with my everyday life and not feeling debilitated in any sense. *(016 BC)

*It's feeling joyful about the world, I think, you want to go out and do things. *(019 BC)

*That you're more in control of what's happening to you. *(018 BC)

While others had difficulty expressing what the concept of wellbeing meant to them, subsequent descriptions of how they felt their lives had changed as a result of acu/moxa treatment revealed that they perceived it to have had a substantial and positive impact on aspects of their physical and psychosocial health.

#### 3.4.1. Physical Changes Associated with Acu/Moxa

Improved energy levels were experienced by many participants. For some, this was merely a short-term effect that dissipated, “as though you've charged your battery up and it gradually discharged down again” (009 HNT). For others, the effect was more enduring:

*The first time I went home and I was bounding, you know. Because *[name of acupuncturist]* had said that I might feel quite tired and a bit drained and I didn't … I felt very energetic … I just felt woken up, you know, much more energetic. Which has continued, to be honest. *(002 BC)

One woman (005 BC), whose energy levels had been so low that she needed to go to sleep when she got home from work at 5.30 pm, reported a continuous improvement following the third treatment. As a result, she was able to stay awake and be active until 9 p.m. and later. For another, the renewed energy was linked closely with her general motivation to do things and a sense of things returning to “normality”:

*Now getting up in the morning I have energy all day, whereas before I was on the couch in front of the telly, I couldn't be bothered … now I'm back and I'm doing, you know, my everyday life is normal. I feel like I've been given my life back. *(016 BC)

Improvements in sleep patterns were also attributed to the acu/moxa treatments. For some, sleep that had been disturbed by other physical symptoms, such as chronic itching, night sweats, or chest infections, improved markedly when those symptoms were relieved. For others, sleep patterns that had gradually deteriorated for no apparent reason improved markedly after starting acu/moxa. One woman, who had suffered from recurrent nightmares since her cancer diagnosis ten years previously, found acu/moxa very effective in this respect:

*I've had terrible nightmares, to the point that I was frightened to go to sleep … I'd wake up screaming, crying, sweating … and it's been like that ever since *[the diagnosis of cancer].* And when I said this to *[name of acupuncturist]* she said ‘we'll work on that' and she did, and I have only had one nightmare since I finished seeing her. Which for me is absolutely amazing, you know. I'm actually getting to sleep. *(003 BC)

Other beneficial physical effects were reported, including relief of musculoskeletal pain, constipation, cold symptoms, thrush, dryness in the mouth, and cramps.

Participants felt able to discuss their physical ailments with the therapists and appreciated that the acupuncturists “could sort of tailor the acupuncture as to your specific needs” (001 BC), even if these were not directly connected to the lymphoedema:

*I also have this ongoing thrush which was driving me bonkers, because I went to the doctor and I had medication and I had the pessaries and I said ‘what's causing this?' and she *[the acupuncturist]* treated that and that went! … I just mentioned it on the off chance and she said ‘I have a remedy for that' and I said ‘well you could try it' and it worked, I was so pleased. *(005 BC)

The study was not designed to specifically address the symptoms associated with lymphoedema itself, such as reducing swelling. However, some participants felt acu/moxa treatment had contributed to a reduction in lymphoedema-related discomfort. Many perceived that swelling decreased; others spoke of reduced sensations of heaviness and discomfort and increased mobility and function. Sometimes this lasted only for the duration of the course of acu/moxa treatments:

*Yes, as I said, when I was having acupuncture, I could move my arm and my shoulders, and then when it seized up again I had to go back to the tablets to be able to do it … it gradually wore away. *(008 HNT)

In some cases, a more long-term relief, especially from pain, was experienced. Such pain relief had a knock-on effect, improving overall quality of life and the ability to cope with chronic health issues:

*… I had dreadful pain in my elbow and in my wrist, and each treatment seemed to improve it. And by Christmas the pain had gone, and that felt wonderful, because I think without the pain I felt more positive, I didn't have to take the painkillers, you know, I actually stopped taking the pain killers by Christmas and I haven't gone back to them, I haven't taken any painkillers since Christmas. And I mean really and truly, it's just maybe being able to cope with it better because it definitely doesn't rule me as it did. *(004 BC)

#### 3.4.2. Psychosocial Changes Associated with Acu/Moxa

Some women, particularly those with family commitments, expressed the view that attending the acu/moxa sessions offered them the opportunity to have time for themselves, which they valued highly. A large number of participants spoke of acu/moxa being relaxing and a means of helping them deal with stress and chronic anxiety:

*I was quite an anxious person, and I found it helped me to relax and feel better in myself, not so anxious. It did help immensely, really. *(021 BC)

Once again, for some these benefits were short-term while others felt acu/moxa conferred a long-term benefit and a change in outlook:

*I've had a lot of stress from outside, all the time I was having the treatment, and I'm sure that it helped me, you know, to cope with it. Because since the treatment stopped I know I haven't been coping with the stress so well. *(010 HNT)

*Yes, well for me it was feeling relaxed, because as I say I'm always tense, and I would worry and I agonise over things and I definitely felt more relaxed. And in fact I feel better now than I did during the sessions. I seem to be having a delayed reaction. *(006 BC)

The therapists also fulfilled what participants called a “counselling role” during the sessions, giving them the opportunity to talk and to share their problems. Several patients described how they had felt able to talk about their concerns during the sessions; they were able to “offload” and “cry in front of” their acupuncturist. One man found this “counselling” role particularly helpful, as he did not feel it was appropriate to share all of his negative thoughts at home:

*When we have sickness like this, talking at home doesn't generally help. Because it's a depressing thing to talk. But when you're talking with somebody like *[name of acupuncturist]* or here, it's more professional and at the same time you are able to tell them what you would otherwise not want to unnecessarily depress the rest of the family. So you are very open, and she is receptive, and she is also counselling, because that part is very important, besides the needles, the counselling part, and then supported by the acupuncture and everything else. *(007 HNT)

(It is important to note that the term “counselling” is one used by the participants and not by the acupuncturists. While the acupuncturists had been trained in active listening skills, at no time did they promote themselves as counsellors.)

### 3.5. Empowerment, Control, and Acceptance

Descriptions of how participants felt their lives had changed because of acu/moxa revealed that they perceived it to have had a substantial and positive impact on their wellbeing. The capacity to enjoy an active life, without feeling debilitated, and to feel motivated and in control was important to these individuals. For many, acu/moxa treatment facilitated the progress from being disabled by lymphoedema to enjoying enhanced wellbeing and improved quality of life, giving individuals a sense of empowerment and control. These benefits extended beyond the individual to affect family and social relationships:

*And I think having the acupuncture helped me to find myself and actually say to the family ‘I'm sorry, I'm tired, my arm hurts, either do it yourself or leave it 'til tomorrow!'. And the funny thing was the roof did not fall in, and people did still speak to me. But I'd lost that confidence and people keep saying that I'm like my old self now, I can't tell you what it means. *(003 BC)

* I just feel so much more in control of myself. I feel a happier person and I've got far more time, far more patience with the grandchildren, you know. We go out and we do things far more now than what we used to do, even this time last year I didn't have the energy that I have now, it's marvellous. *(004 BC)

Some participants, who had been particularly traumatised by the experience of cancer and other personal problems, described the acu/moxa treatment as a life-changing experience:

*It gave me so much confidence, I even changed my job! Which was incredible under the circumstances, you know, and I've done so well with my job … I just started feeling so much better after the acupuncture. And I've gone back full-time, I was only doing part-time at the time, so all-in-all I've really got my life back. *(017 BC)

Several participants spoke of a sense of being back in control of their lives after the debilitating experience of being diagnosed and treated for cancer:

*I think the underlying feeling is that you are actually able … you're doing something to help yourself, where prior to doing this, you felt quite useless. There was nothing you could do to aid your recovery, really. *(018 BC)

*The biggest thing that it's done for me is to put me back in balance … it *[lymphoedema]* doesn't let you forget the cancer I think, because it's a physical reminder of the fact that at one point in your life you were so very vulnerable. So it's a bit like a monkey sitting on your shoulder, most of the time he's on your shoulder but every now and then he comes and slaps you in the face. I just feel I can slap him back now, you know. *(003 BC)

Acu/moxa could also be a catalyst that generated the motivation to take a more active part in self-care, necessary to manage a chronic condition like lymphoedema:

*When I came back in January … I said to her ‘I've joined Weight Watchers, I'm really being positive about this and I'm really working on my exercises for the lymphoedema, I'm really working hard' … and I honestly say this is the acupuncture and the moxibustion … it changed the way I thought about myself. *(004 BC)

Others experienced a more open and positive approach to life in general, replacing a sense of lethargy and lack of motivation:

* It was just a general feeling of wanting to get on and try something new and do something, test out a new little project you'd had in mind for some time, rather than ‘oh, I might do it tomorrow', which was more the attitude previously. *(019 BC)

In some cases, even when there had been no discernible change in the condition, participants felt that having acu/moxa had reduced their concern about the lymphoedema and enabled them to tolerate it more:

*I think we were always made aware from the very beginning, that it wasn't going to cure lymphoedema, and I was always aware of that and she always said ‘this is to help the surrounding things that are in your life, that will aid the lymphedema'. And I think we've had such incredible results from it for other things, that it almost overshadowed what was happening with the lymphoedema … It wasn't such a dominating factor in your life. *(018 BC)

*You've got to learn to live with it, and as you say, what this does, it helps you to get on with life really, accepting it as what you've got. *(009 HNT)

### 3.6. Degree of Change Attributed Specifically to Acu/Moxa

Within the groups, there were discussions about whether it was the acu/moxa per se or the time and attention they received that had led to the emotional and psychosocial benefits. Several participants said “I wasn't expecting anything,” so they were “delighted when something happened” (018 BC, 019 BC).

One participant was medically trained and had been interested to find that her knowledge did not really explain the experiences she had during the acu/moxa treatment:

*I was enormously interested because the sensations that I got didn't bear any resemblance to the nervous system that I'd learned as a physio. And that didn't make sense, so I thought ‘there must be something here that Western medicine doesn't accept'. *(014 BC)

In general, the participants felt that it was unimportant to know what had caused them to experience benefits:

*All the time I was having it, I read several articles in the papers with doctors saying acupuncture doesn't work, and well, it worked for me, and whether it was in my mind or not, it works! … It doesn't matter! *(010 HNT)

*And as I said, if it is all in the mind I don't care! It worked! *[sounds of general agreement with this]* And I don't believe it is anyway. *(003 BC)

## 4. Discussion

### 4.1. Summary and Comparison with Existing Literature

#### 4.1.1. Perceived Experiences and Effects of Treatment

Participants in this study initially regarded acu/moxa with some scepticism or as a last ditch attempt to improve their wellbeing when other therapies had failed. However, after treatment participants reported a range of changes to their overall wellbeing, most of which were positive, many of which were unexpected. Physical changes included improved energy levels and mobility, better quality of sleep, and reduced pain, as well as improvements in a range of other somatic symptoms. In some cases, symptom reduction meant medication could be reduced. Among the psychological benefits, alleviation of stress and chronic anxiety were reported as well as an increased ability to relax, which was highly valued.

Although not experienced by every participant, the emerging themes from these focus groups suggest that acu/moxa treatment enabled a transition from the sense of disempowerment brought about by cancer diagnosis and treatment and the associated sequelae, to a state of empowerment. Many participants described new feelings of control, confidence, and a sense of balance in their lives. Such changes enabled improved social functioning, such as in family relationships and the workplace.

This wide range of reported outcomes associated with traditional acupuncture treatment is similar to those found in other qualitative studies [44–47]. The perceived benefits reported here are also supported by the quantitative findings of the trial [23], which demonstrated clinically and statistically significant improvements in individualised health status as measured by the Measure Yourself Medical Profile (MYMOP) [48, 49]. Participants in the overall study had experienced a wide range of troublesome symptoms including anxiety, stress, feeling depressed, poor sleep, musculoskeletal problems, and other somatic symptoms. The cooccurrence of multiple symptoms in these participants is consistent with the symptom burden reported in both short- and long-term cancer survivors, with the consequent impact on quality of life [50–52].

Of particular interest are the perceived changes in lymphoedema-related symptoms which were the most frequently specified bothersome symptom on the MYMOP questionnaires [23]. Although it was clearly stated in the study documentation that acu/moxa treatment was not intended to treat lymphoedema itself, many participants reported improvements in their lymphoedema-related symptoms, including increased mobility and reduced sensations of heaviness, pain, and other discomforts. Many perceived decreases in swelling, although they were disappointed when measurement by the lymphoedema nurse specialist showed no significant changes in volume. Although this might suggest that participants merely experienced a shift in their perception of the problem rather than an actual physical change, this appeared to confer a substantial emotional and psychological benefit. Furthermore, the importance of reducing lymphoedema-related discomfort is highlighted by results of another study which found that, for breast cancer survivors, swelling and severity of lymphoedema were less correlated with quality of life than arm symptoms and pain, leading to the recommendation that clinicians should focus on arm symptoms and pain “as much if not more than arm swelling” [53].

Also of interest are the participants' descriptions of increases in their energy levels, a benefit that is often reported anecdotally by acupuncturists and increasingly reported in research [46, 47, 54].

These qualitative reports of changes in lymphoedema-related symptoms and in energy are supported by the quantitative data from the main study. Analysis of the SF-36 health survey questionnaire [55] showed significant improvement on the Bodily Pain and Vitality scales for breast cancer participants at all measurement points up to and including four weeks after the end of treatment [23].

#### 4.1.2. Using Acu/Moxa in the Management of Lymphoedema

The participants in this study valued the whole-person approach of the traditional acupuncturists. That they were listened and responded to and that all aspects of their health were taken into consideration were of particular importance to the majority of participants, as was feeling cared for. In addition, they placed a high value on the fact that treatment was tailored to their individual requirements. These factors were also reported as being important to acupuncture patients in a range of other studies [44–46, 54].

**Figure 1:**
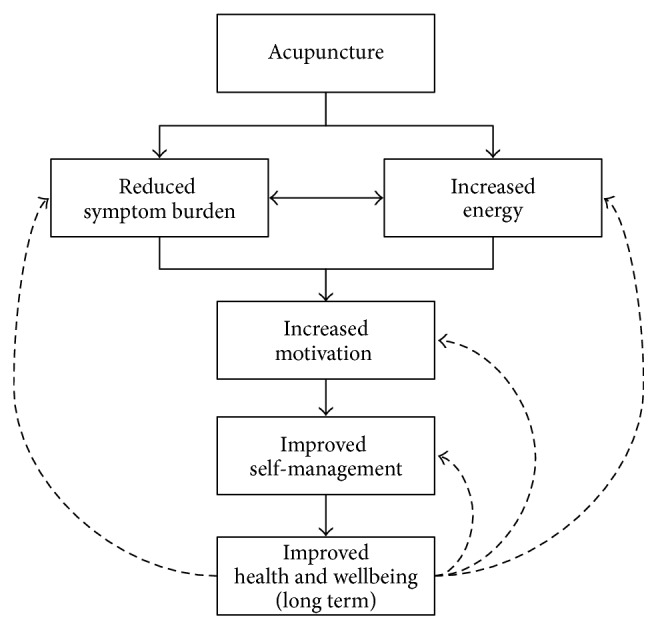
Acu/moxa as a process for long-term healthcare.

In a study of early breast cancer patients undergoing chemotherapy, Price et al. [47] report how these combined characteristics of traditional acupuncture treatment contribute to “enabling coping”: that alleviation of symptoms and improved wellbeing lead to an improved mental outlook and increased ability to cope with cancer diagnosis and chemotherapy treatment. The emerging themes from this qualitative study echo those of Price et al. and also include the dimension of improved self-care, a necessary aspect of managing chronic conditions such as cancer and lymphoedema [1]. Building on observations made in our work with cancer survivors [23, 56] we propose a model illustrating the potential for acu/moxa to improve long-term health (Figure 1).

Participants in this study described a reduced symptom burden and improved energy levels as perceived benefits of their acu/moxa treatment. These outcomes feed into each other and in turn have the potential to improve motivation and self-care. This has a particular value for people with lymphoedema, as successful management of their chronic condition is crucially dependent on the quality of their daily self-care. In this way the potential outcome is improved overall wellbeing, which has a further potential of generating future cycles of improved self-care and enhanced wellbeing and thus the overall long-term health of the patient. It is important to regard acu/moxa as a process that enables transformation and improvement in overall wellbeing, rather than a magic bullet targeted at a specific symptom [57].

There is strong evidence to support the model set out in Figure 1. Participant 004 BC (quoted above) provides the clearest example of this, as she reports that acu/moxa treatment has been a catalyst for her enhanced self-care including her motivation to lose weight and to do her daily exercises. Others describe a more subtle transformation, reporting that improved wellbeing led to their increased ability to cope with daily life. The movement through this model can even be illustrated using a within-case analysis, although this was not the stated purpose of this study. One particular participant (003), for example, had experienced positive effects on her symptoms:

*At one point I couldn't get away from the lymphoedema because it was such an effort just to lift the arm, and it's not like that now. And I've got movement back. *

Her motivation appeared to have increased, both in terms of being able to manage family commitments and in terms of protecting and caring for herself:

*I mean I've got six grandchildren now but I'm now starting to look after my daughter's six month baby. I couldn't have done that last year …. I've actually found it's given me a much better attitude towards the lymphoedema … And I think having the acupuncture helped me to find myself and actually say to the family “I'm sorry, I'm tired, my arm hurts, either do it yourself or leave it 'til tomorrow!”. *

She spoke very positively of her experience of acupuncture and her new sense of wellbeing was palpable:

*But I'm just so grateful I had it, I can't say thank you enough, it's made such a difference to me … I'd lost the ability to laugh at myself, and I think once you lose that it's worrying isn't it. I can laugh now.*

### 4.2. Strengths and Limitations

This qualitative study has a number of strengths. It is the first qualitative study to explore lymphoedema patients' perceptions of acu/moxa treatment. Furthermore, it addresses overall wellbeing, rather than focussing on a single physiological symptom (usually reduction in swelling in lymphoedema studies). It also includes head and neck cancer survivors with lymphoedema, an underresearched group of cancer survivors.

Another strength of the study lays in the varied experience and expertise of the research team. As the qualitative researcher carrying out the focus groups (Anthea Asprey) had little knowledge either of acupuncture treatment or of lymphoedema, there was therefore a need for focus group participants to give very full accounts of their experiences and responses, resulting in very rich data. The expertise of the other two researchers in this study added insight into the interpretation of the data: Teresa Young has extensive experience of research into the supportive care of cancer survivors, whilst Beverley de Valois is an experienced acupuncturist and researcher with a specialist interest in issues of cancer survivorship including lymphoedema.

The study was restricted to English-speaking participants, and there was a lack of representation of the diverse ethnic population in the geographical area. However, considerable diversity in age, educational level, and duration of lymphoedema was obtained. Amongst head and neck cancer survivors, there was equal representation of both sexes.

The small size of the focus groups may have limited the total range of experiences in each group. The planned size of each group was six participants; small groups are easier to recruit and host and are more comfortable for participants [36]. However numerous factors contributed to running smaller groups, and ultimately we aimed for groups of four participants each. These factors included the ability of participants to attend on the dates that other resources were available, such as the qualitative researcher and meeting rooms at the hospital. Last minute cancellations and no shows, due to illness or work commitments, also impacted on group size, with one group reduced to two participants at short notice. Our decision on this occasion was to proceed with the focus group, even though it was small.

A further limitation is the lack of data about participants' concerns about safety. People with lymphoedema are advised to avoid accidental and nonaccidental skin puncture in the affected area [1] and this raises concerns for patients about the safety of acupuncture. In our study, participants were assured during the recruitment process that needling would be avoided not only in the affected area, but with a wide margin of safety (for breast cancer participants, needling was avoided in the torso as well as the arm on the ipsilateral side). By the time of the focus groups, the participants were accustomed to the idea of having acu/moxa with this wide margin for safety, so it was assumed to be no longer an issue for them.

Finally, it is important to acknowledge that participants who receive treatment in the context of a trial may differ from those attending for usual care.

### 4.3. Implications for Research and Practice

Further research is needed to confirm and add to the findings presented here, which, in conjunction with the quantitative aspects of the entire study, are a necessary preliminary to conducting a randomised controlled trial. Aspects to consider in future study design include investigating the effects of combined acu/moxa as opposed to the more common focus on acupuncture needling alone. In view of the complex, multiple morbidities experienced by cancer survivors, the focus of research should encompass overall wellbeing, rather than isolating single symptoms, most specifically arm swelling in breast cancer survivors. As recommended by Price et al. [47], there is a need to develop research methods and tools that measure the whole-person treatment approach and effects for participants over time. In addition, further investigation into the role of acu/moxa in facilitating improved self-care is required.

## 5. Conclusions

This qualitative study indicates that acu/moxa has the potential to benefit some people with cancer-related upper body lymphoedema, who present with a number of symptoms related to, and in addition to, lymphoedema. Perceived benefits include physical and psychosocial changes that may be independent of the presenting complaint and may encompass improved self-care leading to longer term health improvement. Participants valued the aspects of traditional acu/moxa treatment, and many reported that it facilitated a transformation from the disempowerment of cancer diagnosis and the consequences of treatment to feeling empowered and in control of their lives.

## Figures and Tables

**Table 1:** Focus group details.

Focus group	Planned number	Actual number	Gender	Cancer type	Nonattendees	Duration (minutes)
1	4	4	1 female 3 male	4 HNT	0	62
2	4	2	4 female	4 breast	2^1^	42
3	4	4	4 female	4 breast	0	56
4	5	4	5 female	1 HNT 4 breast	1^2^	51
5	4	4	4 female	4 breast	0	70
6	5	4	5 female	1 HNT 4 breast	1^3^	72

^1^1 participant cancelled at short notice citing work commitments; 1 participant cancelled at short notice, explaining she felt unable to participate in a group but would like to give feedback. A telephone interview was arranged to accommodate this.

^2^1 participant was unable to come to this group and was rescheduled to focus group 6.

^3^1 participant felt ill and was unable to attend at short notice.

**Table 2:** Questioning route (abridged).

Introductory	How did you learn about this research study?
Transition	Think back to when you first became involved in the study: (i) How did you feel when the lymphoedema nurse first suggested you try acu/moxa? (ii) How well did your first meeting with the research acupuncturist prepare you for your involvement in the study?

Key	What was it like for you actually having the treatments? What does the term wellbeing mean to you? How has your wellbeing been affected as a result of acu/moxa treatment? How has the treatment affected your attitude to lymphoedema? How do you feel about the commitment that taking part in research involved?

Ending	What improvements could we make for a future study?

**Table 3:** Participant characteristics.

Characteristic	HNC, *n* = 6	BC, *n* = 17	Total, *n* = 23
*Age, years*			
Mean [minimum–maximum]	63.7 [50–83]	58.5 [43–73]	59.9 [43–83]

*Gender*			
Female, *n* [%]	3 [50]	17 [100]	20 [87]
Male, *n* [%]	3 [50]	0 [0]	3 [13]

*Ethnicity*			
White *n* [%]	5 [83]	15 [88]	20 [87]
Asian *n* [%]	1 [17]	2 [33]	3 [13]

*Education*			
Less than compulsory	2 [33]	0	2 [9]
Compulsory	2 [33]	4 [23]	6 [26]
Postcompulsory	0	8 [47]	8 [35]
University	2 [33]	3 [18]	5 [22]
Postgraduate	0	2 [12]	2 [9]

*Employment*			
Retired	5 [83]	6 [35]	11 [48]
Not employed	0	3 [18]	3 [13]
Working part-time	0	5 [29]	5 [22]
Working full-time	1 [17]	3 [18]	4 [17]

*Duration of lymphoedema, months*			
Mean (SD)	60.00 (46.63)	46.76 (31.88)	50.22 (35.62)
Minimum–maximum	6–108	12–132	6–132

*Months from surgery to acu/moxa tx*			
Mean (SD)	74.17 (42.56)	93.71 (81.55)	88.61 (72.97)
Minimum–maximum	16–131	6–278	6–278

HNC: head and neck cancer, BC: breast cancer, SD: standard deviation, and tx: treatment.
